# Tetraspanin 15 depletion impairs extracellular vesicle docking at target neurons

**DOI:** 10.1002/jex2.113

**Published:** 2023-09-11

**Authors:** Daniele Stajano, Franco L. Lombino, Michaela Schweizer, Markus Glatzel, Paul Saftig, Kira V. Gromova, Matthias Kneussel

**Affiliations:** ^1^ Institute of Molecular Neurogenetics, Center for Molecular Neurobiology, ZMNH University Medical Center Hamburg‐Eppendorf Hamburg Germany; ^2^ Core Facility Morphology, Center for Molecular Neurobiology, ZMNH University Medical Center Hamburg‐Eppendorf Hamburg Germany; ^3^ Institute of Neuropathology University Medical Center Hamburg‐Eppendorf Hamburg Germany; ^4^ Biochemical Institute Christian‐Albrechts‐University Kiel Kiel Germany

**Keywords:** docking, exosome, extracellular vesicle, intercellular communication, neuron, target cell, tetraspanin

## Abstract

Neurons in the central nervous system release extracellular vesicles (EVs) and exosomes in response to synaptic activity to regulate physiological processes at target neurons. The intercellular transfer of proteins, mRNAs, lipids or metabolites through EVs potentially modulates the structure and function of neurons and circuits. Whereas the biogenesis of EVs, their release from donor cells, and their molecular composition have been studied extensively, the critical factors and mechanisms regulating EV interactions with target cells are incompletely understood.

Here, we identified tetraspanin 15 (Tspan15) as a component of tumor susceptibility gene 101 protein (TSG101)‐ and CD81‐positive EV fractions. Tspan15 fluorescent fusion proteins were released from donor cells and interacted with target cells together with the exosomal marker CD63. EVs collected from wildtype cortical neurons (WT‐EVs) underwent similar association with target neurons derived from either wildtype (+/+) or Tspan15 knockout (−/−) mice. In contrast, target cell interactions of EVs collected from Tspan15 (−/−) cortical donor neurons (KO‐EVs) were significantly impaired, as compared to WT‐EVs. Our data suggest that Tspan15 is dispensable at target neuron plasma membranes, but is required at the EV surface to promote EV docking at target neurons.

## 1 INTRODUCTION

Extracellular vesicles (EVs) constitute a group of membranous structures secreted by almost every cell type. They comprise exosomes and microvesicles, which originate either from the endosomal system or the plasma membrane, respectively (Budnik et al., 2016; Gurung et al., 2021; Schnatz et al., 2021; van Niel et al., 2018). EVs are present in various biological fluids, regulate multiple physiological processes and contribute to different diseases (Xiao et al., 2021). In general, EVs mediate a specific mode of intercellular communication that enables cells to exchange genetic material, proteins and lipids (van Niel et al., 2018).

In the nervous system, neuronal communication and network function require an interactive exchange of molecules between pyramidal neurons, interneurons and glial cells including astrocytes, oligodendrocytes or microglia (Budnik et al., 2016). Notably, the EV‐mediated shuttling of reciprocal signals between myelinating glia and neurons can promote neuronal survival (Frohlich et al., 2014). Furthermore, exosomes are capable of transferring enzymatic activities to recipient cells, thereby increasing neuronal firing rates (Budnik et al., 2016; Frohlich et al., 2014).

With respect to pathophysiological processes, EVs have been identified as critical mediators in the spread of neurodegenerative disorders, such as Alzheimer´s disease (Freese et al., 2010) or brain cancer (Zhang & Wrana, 2014). In neurons, prion disease onset is accelerated following injection of pathogenic prions into knockout mice of the trafficking factor muskelin, which controls exosome release through multivesicular bodies (MVBs) (Heisler et al., 2018). Moreover, microglia‐derived extracellular vesicles can contribute to an acute inflammatory response (Bianco et al., 2005; Budnik et al., 2016) that might influence neurotransmitter release by neuronal presynaptic terminals (Antonucci et al., 2012).

Besides their different roles in donor and target cells, EVs mediate physiological functions at extracellular locations. For example, microglia‐derived microvesicles, which contain P_2_X_7_ receptors can be activated by high extracellular ATP levels, thereby causing IL1beta release from these vesicles (Bianco et al., 2005). In addition, exosomes seem to possess the ability to reduce toxic substances such as extracellular amyloid‐beta (Malm et al., 2016).

Proteomic approaches have characterized a large variety of EV‐resident proteins (Quiroz‐Baez et al., 2020), including adhesion molecules, signaling receptors, glycoproteins, antigen presenting molecules and tetraspanins (Gurung et al., 2021). Many EV proteins mediate their action at specific target sites however it is still barely understood which membrane proteins participate in the regulation of EV composition, EV release by donor cells or promote EV docking and/or uptake at target cells.

In general, cells appear to take up EVs through a variety of endocytic pathways that includes clathrin‐dependent endocytosis, caveolin‐mediated uptake, macropinocytosis, phagocytosis or lipid raft‐mediated internalization. Individual uptake mechanism used by a given EV may depend on proteins and glycoproteins found on the surface of both the vesicle and the target cell. However, further research is required to identify and functionally characterize critical transfer factors (Mulcahy et al., 2014).

Tetraspanins (Tspans) are evolutionary conserved transmembrane proteins, which arrange tetraspanin webs at the EV membrane, known as tetraspanins‐enriched microdomains (TEMs) (Boucheix & Rubinstein, 2001). A previous study using non‐neuronal tumor cells reported that exosome‐uptake requires internalization‐prone membrane domains and proposed that TEMs, containing tetraspanin 8 (Tspan8), contribute to target cell selection (Rana et al., 2012). Moreover, an analysis of the TEM interactome suggested that insertion into TEMs may be necessary for protein inclusion into the exosome structure (Perez‐Hernandez et al., 2013), a finding that points to tetraspanins as critical regulators of EV composition and function. Structurally, tetraspanins display four transmembrane domains with intracellular N‐ and C‐terminal tails as well as small and large extracellular loops. Post‐translational palmitoylation of tetraspanin residues regulates their association with TEMs and mediates functional consequences for glutamatergic synaptic function in neurons (Becic et al., 2021; Boucheix & Rubinstein, 2001).

Among the tetraspanin superfamily, tetraspanin 15 (Tspan15) is highly expressed in brain including hippocampal and cortical regions. Tspan15 interacts with the disintegrin and metalloproteinase ADAM10, which plays a major role in ectodomain shedding of neuronal surface molecules with physiological and pathological relevance. In particular, Tspan15 regulates the processing of ADAM10 substrates such as different cadherins, the amyloid precursor protein APP and the prion protein Prp^C^ (Koo et al., 2020; Prox et al., 2012; Seipold & Saftig, 2016; Seipold et al., 2018), the latter of which is known to spread via exosome transfer (Heisler et al., 2018).

Here, we report that Tspan15 is a component of N2a cell‐ and neuron‐derived EVs that interact with target neurons. Since EV target cell contact may depend on the protein composition of either the vesicle or the target cell surface membrane, we applied donor or target neurons from Tspan15 knockout mice, respectively. Our data suggest that EV interactions with target neurons depend on Tspan15 at the EV surface rather than at the target cell plasma membrane.

## 2 RESULTS

### 2.1 Tspan15 is a component of extracellular vesicles derived from cortical neurons

**FIGURE 1:**
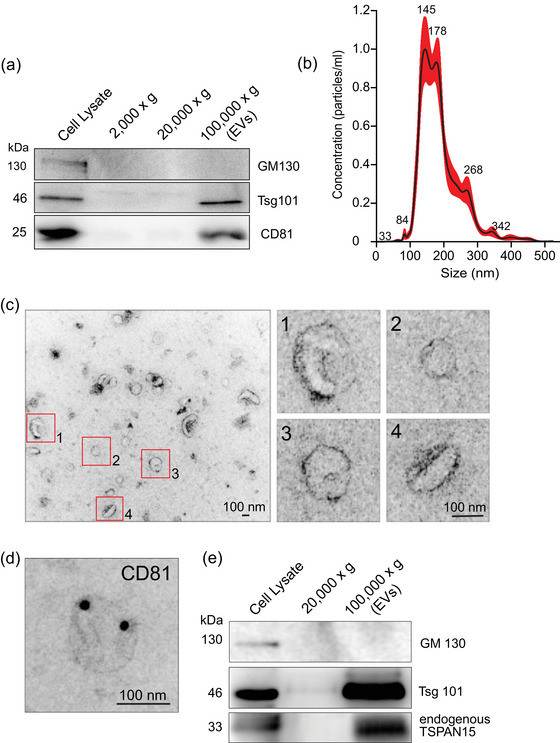
Tspan15 is a component of extracellular vesicles derived from cortical neurons. Analysis of subcellular fractions derived from cultured cortical neurons, following differential centrifugation. (a) Western blot analysis of the post‐nuclear cell lysate, intermediate fractions (2,000 × *g* and 20,000 × *g*) and a EV‐enriched vesicular fraction (100,000 × *g*). Detection of the EV markers CD81 and Tsg101 confirm enrichment of extracellular vesicles (right lane). Detection of the Golgi protein GM130 serves as a negative control. (b) Nano Tracking Assay (NTA) analysis of EV particle sizes within the 100,000 × *g* fraction in A. *N* = 3 experiments. (c) Electron microscopy to visualize the intact membrane of the isolated EVs (negative staining). Red boxes are magnified to the right (scale bars: 100 nm). (d) Immunoelectron microscopy combined with nanogold staining confirms the presence of the EV marker CD81 in the vesicle population shown in C (scale bars: 100 nm). (e) Western blot analysis of subcellular fractions derived from cultured cortical neurons. The EV marker Tsg101 and endogenously expressed tetraspanin Tspan15 are detected in the total cell lysate as well as in the EV‐enriched fraction (100,000 × *g*), confirming that Tspan15 is a component of EVs. Detection of the Golgi protein GM130 serves as a negative control. *N* = 3 experiments.

Several tetraspanin (Tspan) family proteins are components of extracellular vesicles and/or exosomes, including CD9, CD63, CD81, CD82, Tspan 6 or Tspan 8 (van Niel et al., 2018). Since Tspan15 is abundantly expressed in brain and mediates neuronal functions (Brummer et al., 2019; Seipold et al., 2018), we asked whether it might be also a component of EVs derived from mouse cortical neurons. Differential centrifugation of neuronal culture medium derived from neurons at stage DIV (days in vitro) 14, led to enrichment of putative EVs in a 100,000 × *g* fraction. Western blot analysis confirmed that this fraction contained two classical EV markers Tsg101 and CD81, but not the Golgi protein GM130 (Figure 1a). We further validated this fraction by nanoparticle tracking analysis (NTA) using a Nanosight instrument, which revealed an enrichment of particle sizes in the range of 100–300 nm (Figure 1b), characteristic for large exosomes and small microvesicles (Kim et al., 2022; Stahl et al., 2019; van Niel et al., 2018). Electron microscopy confirmed that these particles were vesicles with a diameter of about 100–300 nm (Figure 1c). Using immunogold labelling, we frequently detected the transmembrane protein tetraspanin CD81 at the surface membrane of this vesicle population (Figure 1d). Tsg101‐positive EV fractions, lacking GM 130 (negative control), contained endogenous Tspan15 protein with a molecular weight of 33 kDa (Figure 1e), indicating that Tspan15 is a component of EVs released by cortical neurons.

### 2.2 Subcellular localization of fluorescent Tspan15 fusion proteins

**FIGURE 2:**
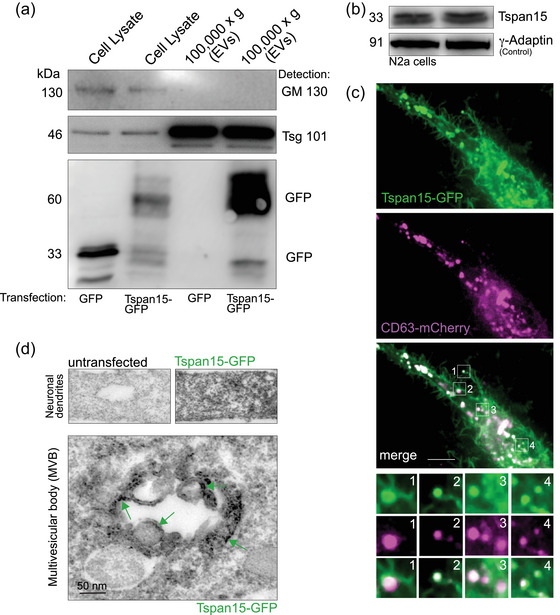
Subcellular localization of fluorescent Tspan15 fusion proteins. (a) Western blot analysis of subcellular fractions derived from N2a cells transfected with either GFP or TSP15‐GFP, following differential centrifugation. The EV marker Tsg101 and Tspan15‐GFP are detected in the total cell lysate as well as in the EV‐enriched fraction (100,000 × *g*). Detection of the Golgi protein GM130 serves as a negative control. Tspan15‐GFP is detected in EV‐enriched fractions. *N* = 3 experiments. (b) Western blot analysis of two independent N2a cell lysates indicating that Tspan15 is endogenously expressed in these cells. Detection of gamma‐adaptin served as a loading control. *N* = 3 experiments. (c) Colocalization of Tspan15‐EGFP (green) and the EV marker CD63‐mCherry (magenta) in N2a cells. Colocalized signals are depicted in white. Boxed‐areas 1–4 are magnified below. *N* = 3 experiments. Scale bar: 10 μm. (d) Immunoelectron microscopy with diaminobenzidine (DAB) using cultured hippocampal neurons expressing Tspan15‐GFP. GFP‐specific antibodies were used for detection. Top left panel: dendritic region of an untransfected control neuron to depict background signals. Top right panel: dendritic region of a transfected neuron expressing Tspan15‐GFP to depict the signal‐to noise ratio. Bottom panel: ultrastructure of a multivesicular body (MVB), representing an organelle involved in EV release. Note the presence of Tspan15‐GFP at intraluminal vesicle membranes.

Since available Tspan15‐specific antibodies do not work for immunostaining, we expressed Tspan15‐GFP fusion protein in N2a cells derived from neuroblastoma. Similar as endogenous Tspan15 (Figure 1e), Tspan15‐GFP was detectable in 100,000 × *g* EV fractions derived from cell culture medium (Figure 2a), represented by an about 60 kDa band. Furthermore, endogenous Tspan15 was detectable in N2a cells by western blotting (Figure 2b). Applying confocal microscopy with N2a cells co‐expressing Tspan15‐GFP and CD63‐mCherry, we found that the majority of Tspan15‐fusion proteins colocalized with CD63‐mCherry that is widely used as a marker for EVs and exosomes (Yoshimura et al., 2016) (Figure 2c, white signals). This prompted us to ask whether Tspan15‐GFP can be found in multivesicular bodies (MVBs), containing membrane‐bound intraluminal vesicles, which represent organelles upstream of EV release (Gurung et al., 2021). To this end, we performed diaminobenzidine (DAB) immuno‐electron microscopy with GFP‐specific antibodies in mouse neurons, expressing Tspan15‐GFP. A comparison of Tspan15‐GFP expressing neurons with untransfected control cells revealed a good signal‐to‐noise ratio (Figure 2d, upper control images). Notably, ultrastructural analysis detected the tetraspanin at the surface of membrane‐bound intraluminal vesicles within multivesicular bodies (MVBs) (Figure 2d, lower image, green arrows).

Together, these data are consistent with the presence of endogenous Tspan15 in neuron‐derived EV fractions (Figure 1e). They add the tetraspanin Tspan15 to the list of potential MVB‐ and EV proteins.

### 2.3 EVs containing Tspan15‐GFP interact with target cells

**FIGURE 3:**
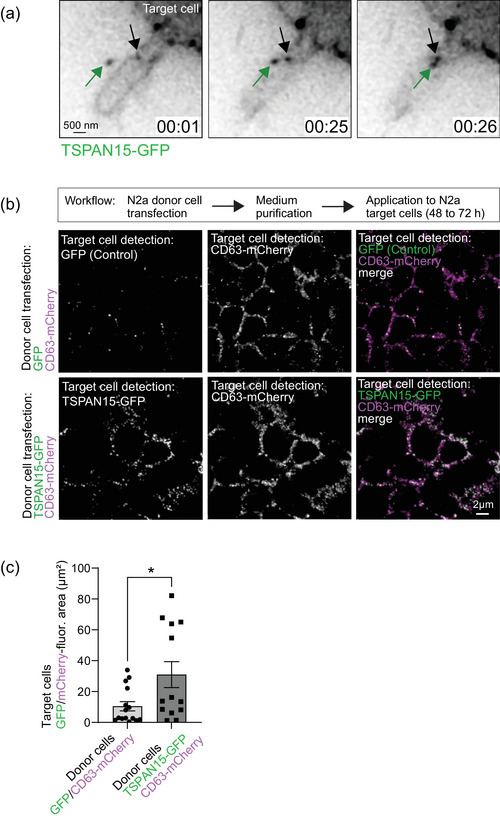
Tspan15 travels with CD63‐positive EVs from donor to target cells. (a) Image derived from time‐lapse video microscopy of Tspan15‐EGFP‐positive vesicles (arrows) derived from N2a cells. An extracellular Tspan15‐EGFP‐positive vesicle (green arrow, left) is moved towards the cell membrane (green arrow, middle) and associates with the cell (green arrow, right), as compared to a static reference vesicle (black arrow) Scale bar: 0.5 μm. (b) Untransfected N2a target cells following incubation with EV‐enriched media derived from N2a donor cells, expressing GFP (upper panels) or Tspan15‐GFP (lower panels) together with the EV marker CD63‐mCherry (magenta). Tspan15‐GFP expressed in donor cells is docking to and detected in target cells. GFP expression served as a negative control. Scale bar: 2 μm. (c) Quantification of GFP/mCherry fluorescent areas (μm^2^) in target cells. Data are represented as means ± SEM (*n* = 13 regions of interest, *N* = 3 independent experiments). Student *t*‐test, *P* value: * *p* < 0.05.

In order to visualize donor cell‐derived Tspan15‐GFP‐positive EVs contacting the plasma membrane of target cells, we performed live cell imaging in neuroblastoma‐derived N2a cells using time‐lapse video microscopy. Tspan15‐GFP vesicles (Figure 3a, left, green arrow) were recruited to target cells (Figure 3a, middle) and subsequently associated with them (Figure 3a, right), as compared to a static signal (Figure 3a, black arrow).

To further assess whether Tspan15 from donor cells connects to target cells, we co‐expressed either GFP (Figure 3b, upper images) or Tspan15‐GFP (Figure 3b, lower images) together with the EV marker CD63‐mCherry in N2a cells (donor cells). Following purification of the culture medium, putative EVs were applied to target cells over 48–72 h and subsequently analyzed for the subcellular localization of the fluorescent proteins. We found that Tspan15‐GFP connected to a significantly higher extent at the target cell surface, as compared to GFP alone (Figure 3b,c).

**FIGURE 4:**
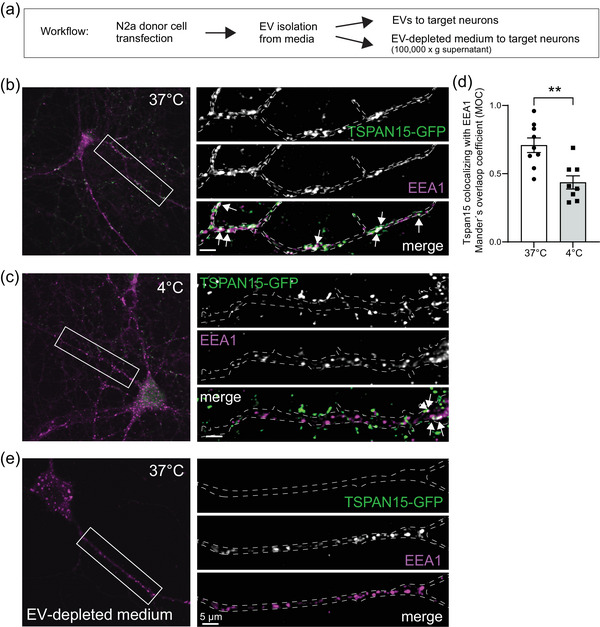
Tspan15‐GFP fusion proteins derived from N2a donor cells colocalize with EEA1 in target neurons. (a) Schematic representation of workflow. (b, c) Tspan15‐GFP fusion proteins (green) derived from donor N2a cells are detected in untransfected target neurons at 37°C (b) or 4°C (c). In the merged panels, white arrows depict Tspan15‐GFP in colocalization with the endogenous early endosome marker EEA1. Scale bars: 5 μm. (d) Analysis of Tspan15‐GFP colocalization with EEA1 expressed as Mander's overlap coefficients. (e) Incubation of EV‐depleted medium at 37°C served as negative control. *N* = 3 independent experiments, *n* = 12 neurons (37°C); *n* = 8 neurons (4°C). Student's *t*‐test of Mander's overlap coefficients. *P* value:* * *p* = 0.0018.

In order to assess whether Tspan15‐GFP‐positive EVs just decorated the outer target cell surface or further underwent internalization, we incubated EVs with cortical neurons and subsequently analyzed Tspan15‐GFP together with the early endosome marker EEA1 (Figure 4a). Tspan15 signals (green channel) to some extent colocalized with EEA1‐positive endocytic vesicles (magenta channel) (Figure 4b, white puncta in merge, arrows). Interference with EV internalization through a temperature drop to 4°C (Yumoto et al., 2012) significantly reduced Tspan‐GFP/EEA1 colocalization (Figure 4c,d), which was undetectable in EV‐depleted control conditions (Figure 4e). Together, we conclude that Tspan15‐positive EVs can interact with target cells and can be partially internalized, however EV internalization through an endocytic or phagocytic mechanism might still be independent of the tetraspanin.

### 2.4 Tspan15 expression is dispensable at target neurons for EV‐plasma membrane interactions

**FIGURE 5:**
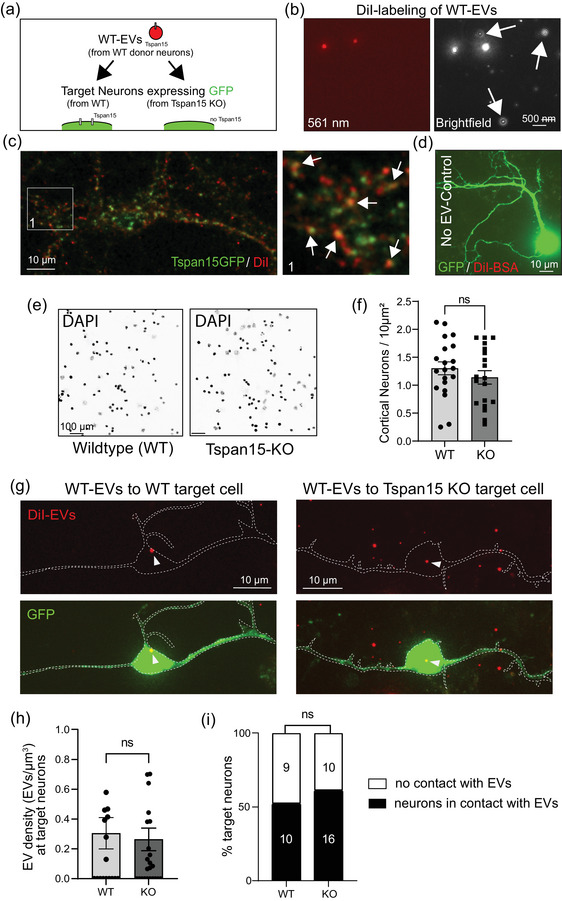
Tspan15 expression is dispensable at target cortical neurons with respect to EV interactions. (a) Schematic representation of the experimental design. Equal amounts of DiI‐labelled fluorescent extracellular vesicles (red) from wildtype (WT) donor neurons were added to the culture medium of cortical target neurons transfected with GFP (green) derived from either wildtype (WT) mice or Tspan15 knockout (KO) mice. (b) Nano Tracking Analysis (NTA) imaging of DiI‐labelled extracellular vesicles from wildtype (WT) neurons. White arrows depict unspecific particles not detected by Dil. Left: detection of fluorescent EVs at 561 nm. Right: detection of total particles with brightfield illumination. Scale bar 0.5 μm. (c) Isolated Tspan15‐GFP‐positive EV labelled by Dil to prove its vesicular character. Scale bar: 10 μm. (d) GFP‐positive cortical neuron incubated with DiI‐treated BSA 0.5% (control), indicating specificity of EV labelling. Scale bar: 10 μm. (e) DAPI staining of neuronal nuclei from wildtype (WT, left) or Tspan15 knockout (KO, right) cultured cortical neurons. *n*(wt) = 19; *n*(KO) = 20 regions of interest; *N* = 3 independent experiments. Scale bar: 100 μm. (f) Quantification of e. ns: not significant. (g–i) GFP‐expressing target neurons (green) derived from cortical neuron cultures. (g) Left: wildtype target neuron incubated with wildtype (WT) EVs derived from wildtype donor neurons. Scale bar: 10 μm. Right: Tspan15 knockout (KO) target neuron incubated with wildtype EVs derived from wildtype donor neurons. Scale bar: 10 μm. Arrows depict Dil‐labelled EVs that underwent interaction with target cells. (h) Quantification of EV density (number of EVs per GFP‐positive volume in μm^3^) in target neurons from g. The Mann–Whitney *U* test was used to assess statistical significance. *N* = 3 independent experiments; wildtype neurons: *n* = 19; Tspan15KO neurons: *n* = 26. ns: not significant. Data are represented as means ± SEM. (i) Quantification of the percentage of target neurons from g in association with EVs. The Fisher's exact test was used to assess statistical significance. *N* = 3 independent experiments; wildtype neurons: *n* = 19; Tspan15KO neurons: *n* = 26. ns: not significant.

We further aimed to compare EV dynamics from wildtype and Tspan15 knockout mice. In a first set of experiments, we collected EVs from medium of wildtype donor neurons (WT‐EVs), with the aim to assess their interaction with target neurons derived from either wildtype or Tspan15 knockout mice (Figure 5a). Tspan15‐GFP‐positive EVs were detected by Diolistic labelling with the lipophilic dye DiI, which fluorescently labels lipid bilayers at 561 nm but is weakly fluorescent until incorporated into membranes. In our hands, Dil did not label unspecific floating particles (Figure 5b, arrows), but specifically labelled Tspan15‐GFP‐positive EVs applied to neurons (yellow signals, arrows) (Figure 5c). No‐EV‐controls using Dil‐BSA further confirmed that the labelling procedure per se did not stain cellular structures of the target cells (Figure 5d). In control experiments using nuclear DAPI staining, we confirmed that the number of target neurons from both genotypes were equal prior to EV application and analysis (Figure 5E,e,f).

Finally, we incubated equal amounts of fluorescent WT‐EVs with target neurons derived from either wildtype or Tspan15 KO mice and assessed the colocalization of Dil‐labelled EV particles with GFP‐labelled neurons using confocal microscopy (Figure 5g). Analysis of XZ and YZ orientations was used to decide whether yellow colocalized particles were either inside the lumen of the cell, in contact with the cell surface or remained outside (not shown). Quantification of EV density at target neurons revealed that EV interactions with the receipient cells were independent of whether the target neuron expressed Tspan15 (WT condition) or not (KO condition) (Figure 5h). Likewise, the percentage of target neurons with WT‐EVs was independent of Tspan15 gene expression (Figure 5i).

We therefore conclude that Tspan15 expression is dispensable at target neurons with respect to EV‐plasma membrane interactions.

### 2.5 Tspan15 expression is required at EV‐releasing donor cells to promote EV interactions with target neurons

**FIGURE 6:**
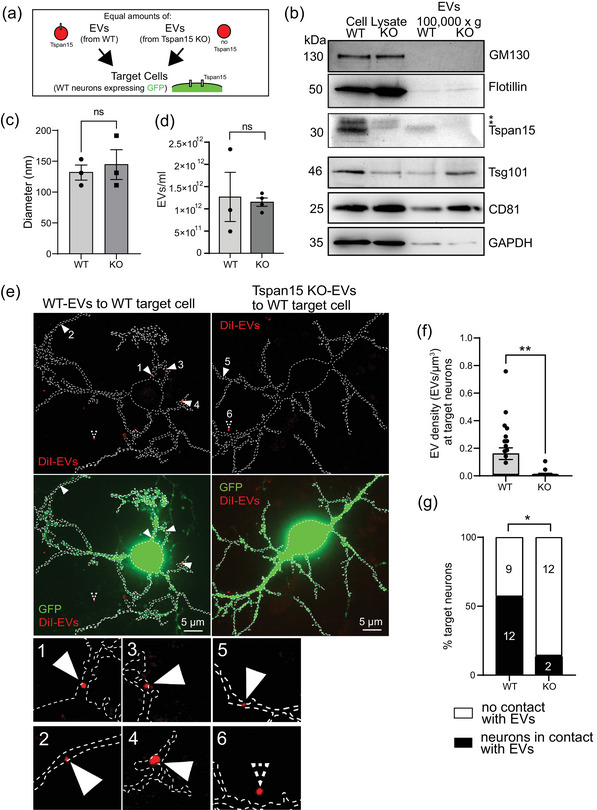
Tspan15 expression is required at EV‐releasing cortical donor neurons to promote EV transfer. (a) Schematic representation of the experimental design. Equal amounts of DiI‐labelled fluorescent extracellular vesicles (red) derived from either wildtype donor neurons or Tspan15 knockout donor neurons, were added to the culture medium of cortical wildtype target neurons expressing GFP (green). (b) Western blot analysis of wildtype (WT) and Tspan15 knockout (KO) extracts. Left: total cell lysates. Right: EV lysates following 100,000 × *g* centrifugation. Detection: GM130 (Golgi marker used as negative control for EVs); flotillin, Tsg101, CD81 (EV marker), GAPDH (loading control). Please note that the Tsapn15 antibody detects three bands above 30 kDa. The two upper bands (asterisks) are unspecific, while the lower 30 kDa band (Tspan15) is not detectable in cell lysate and EV lysate from Tspan15 knockout mice. *N* = 3 independent experiments. (c) NTA analysis of EV diameter. The Mann–Whitney U test was used to assess statistical significance. *N* = 3 independent experiments. ns: not significant. (d) NTA analysis of EV concentrations. Data are represented as means ± SEM. The Mann–Whitney U test was used to assess statistical significance. *N* = 4 independent experiments. ns: not significant. (e–g) GFP‐expressing target neurons (green) derived from cortical neuron cultures. (e) Left: wildtype target neuron incubated with wildtype EVs derived from wildtype donor neurons. Scale bar: 5 μm. Right: wildtype target neuron incubated with Tspan15‐KO EVs derived from Tspan15 knockout donor neurons. Scale bar: 5 μm. Below: magnification of examples depicting Dil‐labelled Tspan15 EVs that underwent transfer to target cells (f) Quantification of EV density (number of EVs per GFP‐positive volume in μm3) in target neurons from e. The Mann–Whitney U test was used to assess statistical significance.  . *N* = 3 independent experiments. Wildtype neurons: *n* = 21; Tspan15 knockout neurons: *n* = 14. P‐value: *p < 0.05. Data are represented as means ± SEM. (g) Quantification of the percentage of target neurons from e in association with EVs. The Fisher's exact test was used to assess statistical significance. *N* = 3 independent experiments. Wildtype neurons: *n* = 21; Tspan15 knockout neurons: *n* = 14. P‐value: *p < 0.05.

In an opposite approach, we asked whether Tspan15 expression in donor neurons might promote EV association with target cells. To this end, we collected extracellular vesicles from medium of either wildtype (+/+) neurons (WT‐EVs) or from Tspan15 knockout (−/−) neurons (KO‐EVs) in order to apply them to wildtype (+/+) target neurons (Figure 6a). Control experiments confirmed that the 100,000 × *g* fractions, enriched for either WT‐EVs (containing Tspan15) or KO‐EVs (lacking Tspan15), contained the EV marker proteins Tsg101 and CD81, but not the Golgi vesicle marker GM130 (Figure 6b). As expected, KO‐EVs did not express the tetraspanin Tspan15, whereas WT‐EVs did (Figure 6b, lower band, note that asterisks marks two unspecific upper bands). The diameters of EVs derived from both genotypes were equal, as assessed by NTA analysis (Figure 6c). We then analyzed the concentration of individual EV fractions (Figure 6d). Due to the variability of EVs harvested from donor cells (Figure 6d), we diluted each fraction accordingly to adapt EV concentrations. NTA measurements further confirmed that equal amounts of EVs were Dil‐labelled from Tspan15 WT and KO neurons (not shown). Consequently, equal amounts of fluorescently‐labelled WT‐EVs and KO‐EVs were applied to target neurons. Remarkably, a significantly higher number of WT‐EVs containing Tspan15 interacted with target cells, compared to KO‐EVs lacking Tspan15, as assessed by 3D confocal image reconstruction (Figure 6e). Neurons lacking Tspan15 gene expression displayed a significantly decreased EV density (Figures 6f). Whereas about 60% of the target neurons had incorporated WT‐EVs, less than 25% of the target neurons contained KO‐EVs (Figure 6g). Although Tspan15‐negative KO‐EVs associated with neurons, their efficiency was markedly reduced. We therefore conclude that the presence of Tspan15 in extracellular vesicles positively affects EV interactions with target cells.

## 3 DISCUSSION

Tetraspanins are membrane proteins with different functions at the surface membranes of cells and vesicles (Budnik et al., 2016; van Niel et al., 2018). Approaches to characterize EV and exosome resident proteins identified different tetraspanins including CD9, CD37, CD53, CD63, CD81, CD82 (Gurung et al., 2021), some of which mediate specific functions in individual cell types while others are very common and can be used as EV marker proteins. Tetraspanins form tetraspanins‐enriched microdomains (TEMs) at the cell surface (Boucheix & Rubinstein, 2001), and may be involved in EV docking and uptake by target cells. Our data add endogenous Tspan15 to the list of EV proteins, as identified in cell culture medium fractions that had been enriched for vesicles of 100–300 nm (Figure 1). Accordingly in donor cells, Tspan15‐GFP fusion proteins are detected in intraluminal vesicles of multivesicular bodies, which represent an organelle upstream of exosome release (Figure 2d).

Using time‐lapse video microscopy (Figure 3a) and transfer assays with temperature drop (Figure 4), we monitored the interaction of individual Tspan15‐positive vesicles with target cells, suggesting that Tspan15 might be involved in cell‐to‐cell communication. EVs can carry a range of nucleic acids and proteins that can have a significant impact on the phenotype of recipient cells (Mulcahy et al., 2014), however it remains to be investigated which factors are components and cargoes of Tspan15‐positive EVs.

Cells take up EVs by a variety of different mechanisms that include clathrin‐dependent endocytosis, caveolin‐mediated uptake, macropinocytosis, phagocytosis, and lipid raft‐mediated internalization (Mulcahy et al., 2014). Tetraspanins might promote these pathways, possibly through mediating interactions with target cell plasma membranes. For instance, Tspan8 promotes exosome uptake into endothelial cells, thereby boosting their activation (Nazarenko et al., 2010).

EVs are of therapeutic interest, as they are disregulated in several diseases (Xiao et al., 2021). EVs could potentially be harnessed to deliver genetic material and drugs to target cells (Zhou et al., 2022) and are potential biomarkers of early disease states (Ollen‐Bittle et al., 2022; Vandendriessche et al., 2022). It is therefore important to characterize the critical factors at the vesicle and recipient cell surface that control EV docking and transfer. In the present study we made use of a Tspan15 knockout mouse to assess interactions of EVs with wildtype target neurons as compared to Tspan15 knockout target neurons. Alternatively, we collected WT‐EVs and Tspan15 KO‐EVs (lacking Tspan15 expression) and compared their interactions with wildtype target cells. Following application of equal amounts of EVs to both genetic conditions, our data suggest that the loss of Tspan15 at the target cell plasma membrane does not affect EV interaction rates. In contrast, we conclude that a loss of Tspan15 in donor cells and consequently at the EV membrane diminishes EV association with target neurons. It is therefore possible that Tspan15 supports interactions and/or docking of extracellular vesicles at target cell plasma membranes. We also show that some EVs undergo internalization as monitored through co‐immunostaining with EEA1, however this downstream process might be secondary and could be independent of the tetraspanin. Although many other membrane proteins could potentially mediate similar functions, Tspan15 regulates the metalloproteinase ADAM10, which in turn is responsible for ectodomain shedding of multiple substrates (Prox et al., 2012; Seipold & Saftig, 2016; Seipold et al., 2018).

Together, our data propose a role of Tspan15 in EV‐mediated cell‐to‐cell communication and suggest that docking of Tspan15‐positive EVs might have the potential to regulate ectodomain shedding at ADAM10‐expressing target cells.

## 4 MATERIALS AND METHODS

### 4.1 Antibodies and DNA constructs

The following antibodies were used: donkey anti‐rabbit Alexa Fluor® 488 (ICC 1:1,000, #711‐545‐152, Dianova), donkey HRP‐conjugated anti‐mouse IgG (WB, 1:15,000, #715‐036‐15, Dianova), donkey HRP‐conjugated anti‐rabbit IgG (WB, 1:15,000, #711‐036‐152, Dianova). Goat anti‐GFP biotin‐tagged (DAB, 1:100, #600‐106‐215, Rockland™), rabbit anti‐biotin (DAB, 1:1000, #100‐4198, Thermo Fisher). Mouse anti‐GAPDH (1:1,000, 6C5, #Gtx28245, GeneTex), mouse anti‐GM130 (WB 1:500, #610823, Biosciences), mouse anti‐TSG101 (WB 1:1,000, 4A10; #GTX70255, GeneTex), rabbit anti‐CD81 (WB, 1:1,000, Nanogold EM 1:200, #10037, Cell Signaling Technology), rabbit anti‐GFP (WB/ICC 1:1,000, #A‐11122, Thermo Fisher). Rabbit anti‐Tspan15 was provided by Prof.Saftig (produced by Pineda). The following constructs were used: pEGFP‐N2 (Clontech), Tspan15‐EGFP provided by Eric Rubinstein (Dornier et al., 2012). CD63‐mCherry provided by Nicole Meisner‐Kober (Corso et al., 2019).

### 4.2 Animals

Tetraspanin 15 knockout mice were generated by Paul Saftig at University of Kiel as described (Seipold et al., 2018). Animals were maintained in the animal facility of the ZMNH, Hamburg (Germany) under controlled environmental conditions. Animal housing complied with all ethical regulations in accordance with the European Communities Council Directive (2010/63/EU), approved by the ethics committees of the city‐state of Hamburg (Behörde für Justiz und Verbraucherschutz, Fachbereich Lebensmittelsicherheit und Veterinärwesen) and the animal care committee of the University Medical Center Hamburg‐Eppendorf.

### 4.3 Genotyping

Genomic DNA of wildtype and Tspan15 knockout mice was extracted from tail or cerebellum biopsies. DNA in Quick Extract Buffer (Biozym Scientific GmbH, Hessisch Oldendorf) was incubated at 65°C for 15 min, following a 2‐min incubation at 95°C. Genotyping was performed via PCR using the following Tspan15 exon 2 primer sequences: 5´‐AAGCTTGGGCTATAGGCATACACC‐3´ and 5´‐ GGATCCCCAGCATCTCTGTGACAGC‐3´.

### 4.4 Cell culture, transfection and immunochemistry

Primary cortical neurons were prepared from embryonic day 16 (E16). Briefly, 12 mm coverslips or 10‐cm plates were coated with poly‐L‐lysine (5 μg/mL in PBS). Brains were extracted and dissected in ice‐cold autoclaved phosphate buffered saline (PBS) supplied with glucose to remove the meninges and to isolate the cortices. To dissociate cells, tissues were incubated in 0.05% EDTA‐Trypsin (Invitrogen) for 5 min at 37°C. The trypsin reaction was blocked by rinsing the dissociated tissues in pre‐warmed Hank's Balanced Salt Solution (HBSS, Invitrogen) supplied with 10% Fetal Bovine Serum (FBS). About 60,000 cells were seeded per coverslip in Lonza PNGM medium (Thermo Fisher Scientific, Dreieich, Germany). N2a cells and primary neurons were transfected with Lipofectamine™ 2000 Transfection Reagent (Invitrogen). For N2a cells, the DNA mix in Opti‐MEM™ (reduced serum medium, Invitrogen) was mixed with Lipofectamine for 20 min. It was then added to cultures at 60% confluence. DIV10‐13 primary neurons were transfected with a DNA‐Lipofectamine mix as mentioned above. The conditioned neuronal medium was saved at 37°C. Primary neurons were incubated for 2 h and subsequently rinsed in pre‐warmed Hepes buffer (10 mM HEPES, 135 mM NaCl, 5 mM KCl, 2 mM CaCl2, 2 mM MgCl2, 15 mM Glucose; pH = 7.4). Eventually, the conditioned media was re‐added to the neurons. For immunochemistry cells were fixed for 7–10 min with 4% formaldehyde/4% sucrose in PBS at room temperature. After fixation, cells were washed three times in PBS and incubated for 1 h at room temperature with primary antibodies diluted in goat serum dilution buffer (GDB) (10% DS, 0,23% Triton X‐100, in PBS). Neurons were then washed three times in PBS (5 min each), following incubation with Cy‐conjugated secondary antibodies in GDB buffer for 1 h at room temperature. After three additional washes in PBS for 30 min each, slides were mounted using Vectashield mounting medium (Thermo Fisher Scientific).

### 4.5 Cell lysates and western blotting

To prepare cell lysates from days in vitro (DIV) 15 cortical neuron cultures the supernatant was aspirated. Cells were rinsed twice in PBS and harvested with a cell scraper. 0.5% Triton‐X, EDTA‐free Complete Protease Inhibitor Cocktail (Roche), in PBS was added. Cells were lysed over 30 min at +4°C on a rotating wheel and subsequently centrifuged at 1,000 × *g* to chieve a post‐nuclear fraction. The resulting supernatant was diluted in 4X Laemmli‐Urea loading buffer and heated for 15 min at 65°C. For western blotting, proteins were separated on custom‐made Acrylamide 4%–20% gradient gels via SDS‐PAGE and transferred to a MeOH‐activated (polyvinylidene difluoride) PDVF membrane via a wet blot or semi‐dry blotting system. The membrane was blocked with 5% milk in Tris‐buffered saline with Tween 20 (TBS‐T: 2 mM Tris‐Base, 150 mM NaCl, 0.01% Tween) and subsequently incubated with the primary antibodies at +4°C in 5% milk TBS‐T either for 1 h or overnight. After TBS‐T washing steps, Horseradish peroxidase (HRP)‐conjugated secondary antibodies were added in 5% milk TBS‐T at room temperature (RT) for 1 h. After a final TBS‐T washing step, membranes were developed with enhanced chemiluminescence substrate (ECL) and imaged at the Intas ECL Chemostar Imager. To remove signals (stripping), membranes were incubated with an acid stripping buffer (25 mM Glycine, 1%SDS; pH = 2.0) for 30 min. Membranes were then washed three times in TBS‐T, blocked again and re‐probed.

### 4.6 Electron microscopy

For electron microscopy and immunogold labelling extracellular vesicles from cortical cell cultures were pelleted by ultracentrifugation at 100,000 × *g* for 70 min. The pellet was resuspended and fixed in 2% paraformaldehyde (PFA). About 5 μL of this solution was adsorbed to glow discharged carbon coated Formvar grids (EMS, Germany) for 20 min. After washing with PBS, samples were postfixed with 1% glutaraldehyde in PBS and incubated on ice‐cold methylcellulose‐uranyl acetate solution for 30 min. Grids were looped out, air‐dried and analysed by electron microscopy.

For immunogold labelling grids with adsorbed EVs were first rinsed in PBS quenched in 20 mM glycine in PBS and blocked with 1% bovine serum albumin (BSA) for 10 min according to (Thery et al., 2006). Thereafter they were incubated with primary antibody rabbit anti‐CD81 (1:200, #10037, Cell Signaling Technology) for 2 h. CD81 was recognized with Protein A coupled to 10 nm colloidal gold particles (G. Posthuma, University Medical Center Utrecht) (1:50).

For DAB staining of Tspan‐EGFP, primary neurons were grown on Aclar foil (Ted Pella) for 10 days. Cells were transfected with Tspan15‐EGFP. Thereafter pre‐embedding immunoelectron microscopy was performed as follows: neuronal cultures were fixed with 4% paraformaldehyde and 0,1% glutaraldehyde in PB. Permeabilization was performed by incubating the sample with EtOH solution as follows: 10% EtOH – 20% EtOH – 40% EtOH – 20% EtOH – 10% EtOH (10 min each). After rinsing in PBS, the coverslips were incubated with 10% horse serum (PS) containing 0,3% bovine serum albumin (BSA) (blocker) in PB for 15 min to block nonspecific binding sites, and incubated with goat anti‐GFP‐biotin (#600‐106‐215; Rockland™) diluted 1:200 in PBS containing 1% PS and 0.2 % BSA (Carrier) over night. The cells were washed with PBS, then incubated with secondary antibody rabbit anti‐biotin (#100‐4198; Thermo Fisher) diluted 1:1000 in carrier for 90 min. After rinsing, they were incubated with ABC (Vector Labs) diluted 1:1,000 in PBS for 90 min. The cells were further washed in PBS, then in 50 mM TRIS and reacted in diaminobenzidine (DAB)‐H202 solution (Sigma) for 10 min. Thereafter the coverslips were rinsed three times in 0.1 M sodium cacodylate buffer (pH 7.2–7.4) (Sigma‐Aldrich) and fixed with 1% osmium tetroxide (Science Services) in cacodylate buffer for 10 min on ice. The samples were dehydrated through ascending ethyl alcohol concentration steps and rinsed twice in pure Ethanol embedded in Epon and polymerized at 60°C for 48 h. All images were acquired with a JEM‐2100Plus Transmission Electron Microscope at 200 kV (Jeol) equipped with a XAROSA CMOS camera (Emsis).

### 4.7 Time‐lapse video microscopy

After transfection, N2a cells were incubated on coverslips. Coverslips were placed in an Attofluor® Cell chamber for microscopy (Thermo Fisher). Images were acquired using a Nikon microscope equipped with the following components: Spinning Disk (Yokogawa) (Visitron Systems), solid state lasers (488, 561, 647 and 405), objectives (60× and 100×), two EM‐CCD cameras (Hamamatsu Photonics 512/1024) containing optical image splitters for simultaneous dual image acquisition, and an incubation chamber for controlled cell culture environment (5% CO_2_ at 37°C). Images were captured at 1–3 s intervals for 30 s.

### 4.8 Isolation of extracellular vesicles and nanoparticle tracking analysis

Conditioned medium of days in vitro (DIV) 15 cortical neurons was harvested and supplied with EDTA‐free complete protease inhibitor (Roche). After centrifugation at 4°C for 10 min at 2,000 × *g*, the resulting supernatant was centrifuged for a second time (30 min, 4°C, 20,000 × *g*) in a swinging‐bucket SW40Ti rotor using an OPTIMA L‐80 XP ultracentrifuge (Beckman Coulter). It was subsequently filtered (0.22 μm filter) and centrifuged for a third time using the same ultracentrifuge setup (1.5 h, 4°C, 100,000 × *g*). The pellet, containing extracellular vesicles, was reconstituted in PBS containing protease inhibitors, which had been filtered with a 0.22 μm filter. Nanoparticle tracking analysis (NTA) was carried out using a NanoSight LM14C (Malvern) setup to achieve the concentration and size of isolated EVs. Capture was performed using a sCMOS camera, and a green laser. Acquisition was achieved using camera level 12, Slide shutter 1200 and Slider Gain of 146. Twenty‐five FPS for a total of 249 frames. Temperature was maintained at 23.9°–24°C. Viscosity was 0.909–0.911 cP as for water. For analysis, detection was performed at the level of 7, the blur size was automatic as well as the maximum jump distance, between 8.0 and 9.1 pixels. The blank level was established by measuring PBS filtered with a 0.22 μm filter. Samples were diluted prior to measurement. For each dilution, 10 recordings were acquired to analyse EV concentration and size. For experiments, equal concentrations of EVs per genotype were used.

### 4.9 EV transfer assay with N2a cells

N2a cells were cultured to 60% confluency in 10‐cm plates following transfection with lipofectamine to express CD63‐mCherry together with GFP or Tspan15‐GFP. After 24 h cells were washed in PBS, detached by pipetting up and down in 6 mL of Opti‐MEM™ (reduced serum medium) and split 1:1 into new plates. After 48 h, the conditioned media containing EVs was centrifuged at 300 × *g* and moved into a new tube. This procedure was performed three times to remove cell debris. The medium was then centrifuged at 3,000 × *g*, filtered with a 0.22 μm filter and warmed up at 37°C. Untransfected N2a target cells, were seeded at low density on 12 mm glass coverslips and were incubated with the EV‐containing medium prepared above. After a 48–72 h incubation time, recipient cells were rinsed with PBS and fixed in ice‐cold PBS supplied with 4% PFA for 7 min. Cells were then washed with PBS and the GFP signal was eventually enhanced by application of anti‐GFP antibodie (Rb, A11122; Abcam). Following mounting with Aqua‐Poly‐Mount, imaging was performed with an Olympus Fluoview FV1000 confocal microscope.

### 4.10 EV internalization assay

N2a cells or DIV15 neurons were incubated for 45 min at 37°C with N2a media‐derived Tspan15‐GFP‐positive EV extract or 100,000 × *g* supernatant (EV‐depleted negative control). The incubation of Tspan15‐GFP‐positive EVs was also performed at 4°C (negative control). Samples were fixed as previously described and permeabilized with 0.1% Triton‐X PBS for 10 min. After rinsing the samples, 1 h blocking in 1% BSA/PBS was carried out at room temperature. Primary antibodies anti‐GFP (Rb, A11122; Abcam) and anti‐EEA1 (Gp, 237 105, Synaptic System) were applied over night at 4°C. After washing with PBS, secondary antibodies donkey anti‐guinea pig Cy5 (706‐175‐148, Jackson Immunoresearch lab.) and donkey anti‐rabbit 488 (711‐546‐152, Jackson Immunoresearch lab.) were applied for 1 h at room temperature. All antibodies were incubated in 1% BSA/PBS. Confocal imaging was performed as reported above. For the analysis of EEA1 colocalization with Tspan15‐GFP the JAKOP ImageJ plugin was used to calculate the Mander´s overlap coefficient.

### 4.11 Analysis of cell density

Cortical cultures derived from wildtype or Tspan15 knockout mice were fixed and stained with 4′,6‐diamidin‐2‐fenilindolo (DAPI) for 10 min and then washed three times in PBS. Samples were mounted on glass coverslips with Aqua Polymount, dried overnight and imaged with an Olympus Fluoview FV1000 confocal microscope. Nuclear regions of interest were quantified as a measure of cell density.

### 4.12 DiI‐labelling and EV interaction assay with neurons

EV extracts derived from primary neuron cultures were validated by either nanoparticle tracking analysis, western blotting or electron microscopy. EVs or 0.5% BSA in PBS (technical control) were labelled for 2 h at 37°C with 0.25 μg/μL of the fluorescent lipophilic dye DiI (Invitrogen) in the dark. Samples were then ultracentrifuged (100,000 × *g* at 4°C, 1.5 h) with a TLA110 rotor (10E30108) using an Optima MAX‐XP ultracentrifuge. The resulting pellets were reconstituted in PBS filtered with a 0.22 μm filter. Wildtype or Tspan15 KO primary cortical neurons were seeded on 12 mm glass coverslips and transfected at DIV 12–13 with a GFP vector. After 24 h, the wildtype and Tspan15 KO neurons were incubated with wildtype DIV15 DiI‐labelled EVs or DiI‐treated 0.5% BSA in PBS (technical control). After 45 min of incubation, the neurons were rinsed with PBS and fixed. Samples were imaged with a Nikon spinning disk confocal microscope, acquiring Z‐stacks of fluorescent neurons (*z* = 1 μm; 100 × objective). Alternatively, DIV14 GFP‐transfected wildtype target neurons were incubated with DiI‐labelled EVs from DIV 14–15 donor neurons derived from either wildtype or Tspan15 KO mice. After 45 min, cells were fixed with ice‐cold PBS supplied with 4% PFA for 5 min. Samples were further processed and imaged as described above. For EV double labelling, N2a‐derived Tspan15‐GFP‐positive EVs were labelled with DiI as described above. Confocal imaging was performed as previously described.

### 4.13 Quantification and statistical analysis

The concentration of EVs was quantified prior to EV interaction experiments. EV concentrations were adjusted to apply equal amounts of EVs to target cells. To quantify EV interaction levels, the number of EVs that were either in contact with the target cell plasma membrane or were detected inside the cellular lumen of GFP‐expressing neurons was assessed. The volume of GFP‐expressing neurons was analyzed using Voxel Counter, an ImageJ (NIH) software plugin. The number of DiI‐labelled EVs was manually annotated using the Cell Counter tool (ImageJ). The analysis was performed on z‐series images, taking into account the relative orthogonal (XZ, YZ) projection views (not shown). EV interaction was calculated as the number of EVs that were found in contact with the GFP‐expressing target cell divided by the cell volume (μm^3^). Statistical analysis was performed with the Prism software (GraphPad, version 9.0.1). If outliers were detected, the ROUT (0.1%) or Grubb (2%) algorithms were applied to the complete datasets. Data are represented  ±  S.E.M. Graphs were generated using the Prism software.

## AUTHOR CONTRIBUTIONS

Daniele Stajano, Franco L. Lombino, Kira V. Gromova and Matthias Kneussel designed the study. Paul Saftig generated the TSAPN15 KO mouse. Franco L. Lombino trained Daniele Stajano in EV isolation techniques. Daniele Stajano, Kira V. Gromova and Michaela Schweizer performed the experiments. Daniele Stajano, Franco L. Lombino, Kira V. Gromova and Matthias Kneussel analysed the data. Daniele Stajano, Franco L. Lombino, Kira V. Gromova, Markus Glatzel and Matthias Kneussel wrote the manuscript.

## CONFLICT OF INTEREST STATEMENT

The authors declare no conflict of interest.
